# Integrative multi-omics quantitative trait loci prioritize CASP7 as a candidate protective gene for cataract

**DOI:** 10.1097/MD.0000000000049709

**Published:** 2026-07-10

**Authors:** Zhengxi Yuan, Xiangyu Ding, Xiaolong Fang, Jingqing Mu, Xia Hua

**Affiliations:** a Aier Eye Hospital, Tianjin University, Tianjin, China; b Aier Academy of Ophthalmology, Central South University, Changsha, Hunan, China; c Aier Eye Institute, Changsha, Hunan, China.

**Keywords:** bioinformatics analysis, cataract, GWAS, pyroptosis, QTL, summary-based Mendelian randomization

## Abstract

Cataracts are the leading cause of vision loss worldwide. Despite surgery being the only effective treatment, its economic burden highlights the necessity of exploring the pathogenesis of cataracts. In this study, we analyzed 4 large-scale GWAS (genome-wide association study) datasets for cataracts and performed SMR analysis along with heterogeneity in dependent instruments (HEIDI) testing to explore the effects of methylation, expression, and protein QTLs on cataracts. We further validated shared genetic variants through COLOC analysis. Additionally, we searched datasets related to cataracts from the Gene Expression Omnibus (GEO) database for differentially expressed genes (DEGs) and Gene Ontology (GO) and Kyoto Encyclopedia of Genes and Genomes pathway (KEGG) enrichment analyses. By integrating summary-based Mendelian randomization (SMR) results with bioinformatics findings, CASP7 showed a consistent protective-direction association with cataract risk (mQTL: OR [95% CI] = 0.959 [0.941–0.977], FDR-adjusted *P* = .039; eQTL: OR [95% CI] = 0.897 [0.860–0.937], FDR-adjusted *P* = .0046; pQTL: OR [95% CI] = 0.597 [0.483–0.738], FDR-adjusted *P* = .00083). GEO-based analyses provided transcriptomic support for CASP7 involvement in cataract-related lens biology. These findings prioritize CASP7 as a genetically supported candidate protective gene associated with cataract risk. Because this study is based on public summary-level and transcriptomic datasets, the results should be interpreted cautiously and require functional validation in human lens-relevant systems.

## 1. Introduction

Cataracts are the leading cause of vision impairment globally, affecting around 79 million individuals aged 50 years or older.^[1,2]^ Although surgical intervention remains the primary treatment, it is associated with high cost and potential postoperative risks.^[3-5]^ Hence, there is a critical need to investigate the underlying mechanisms of cataracts to advance mechanistic understanding and future prevention strategies.

Currently, genetic factors have been identified as significant contributors to cataract development, with twin and family studies suggesting that genetics accounts for 35% to 58% of cataract susceptibility.^[6-10]^ Recent large-scale GWAS and exome-sequencing studies have further expanded the catalog of cataract-associated loci and coding variants.^[11,12]^ For example, Choquet et al conducted a large-scale, ethnically diverse GWAS meta-analysis that identified 54 significant loci. However, GWAS alone has limited ability to pinpoint the genes or molecular mechanisms underlying association signals, particularly when implicated SNPs are located in noncoding regions.

Summary-data-based Mendelian randomization (SMR) integrates GWAS and QTL summary statistics to prioritize genes whose molecular traits are associated with disease through shared genetic architecture.^[13]^ By combining QTL data with GWAS results, SMR can help connect noncoding genetic associations with DNA methylation, gene expression, and protein abundance. However, SMR and colocalization provide genetic evidence consistent with a putative causal relationship rather than definitive biological proof. Multi-omics SMR studies have used blood-derived QTL resources as discovery-stage datasets and then incorporated tissue-relevant datasets for biological interpretation.^[14]^ GEO transcriptomic datasets have also been combined with SMR analyses to provide independent biological context,^[15-17]^ but their interpretation depends on tissue, species, and disease-model comparability. Recent cataract MR studies have begun to evaluate plasma proteins associated with cataract risk,^[18]^ but multi-omics SMR studies exploring cataract pathogenesis remain limited.

Based on GWAS summary statistics, this study proposes a multi-omics framework to identify the mechanisms of cataract. Methylation quantitative trait loci (mQTL), expression quantitative trait loci (eQTL), and protein quantitative trait loci (pQTL) data were integrated to identify the most significantly altered genes, which were subsequently validated in GEO datasets and independent multi-omics stages.

## 2. Materials and methods

### 2.1. Study design

**Figure 1.**
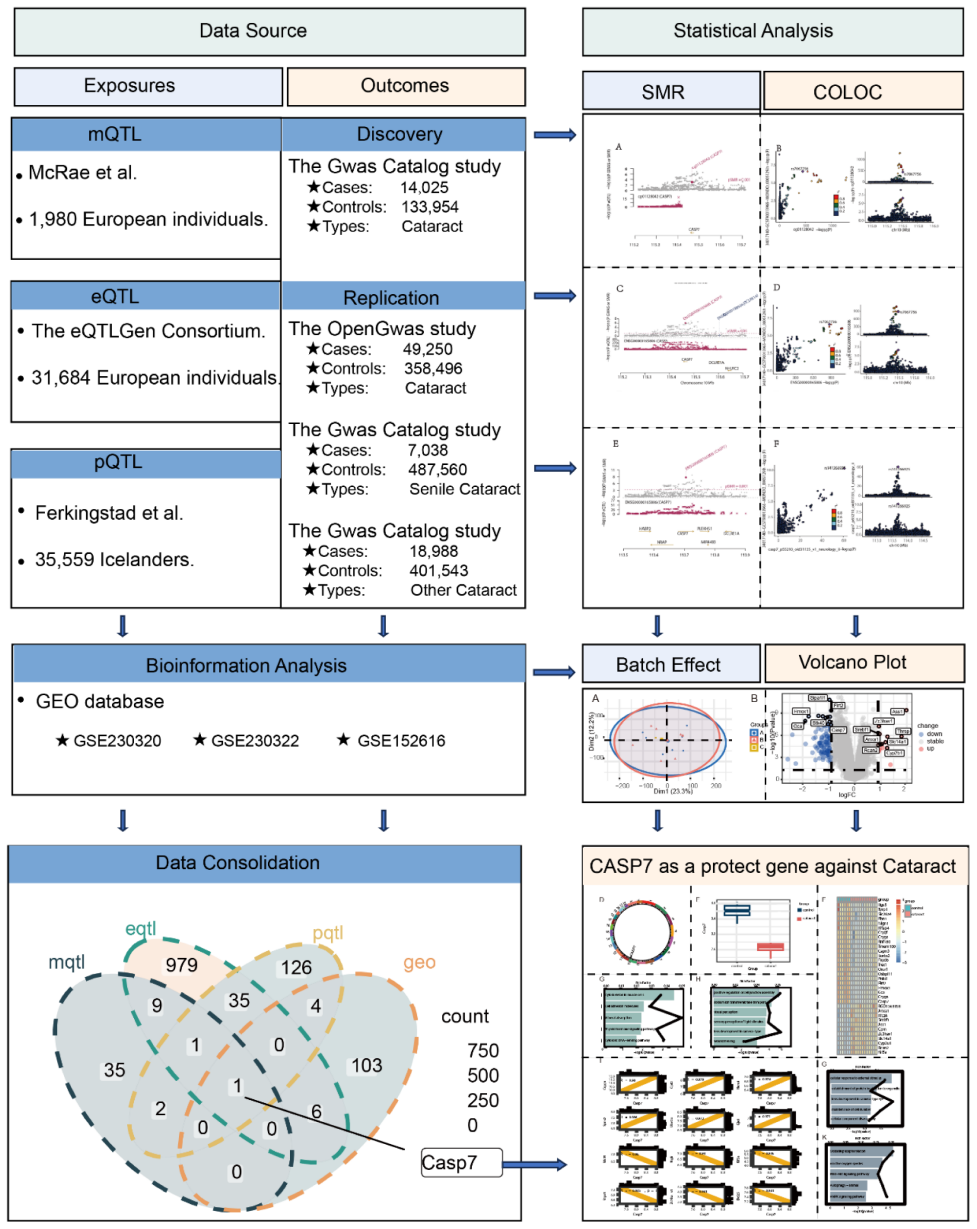
Overview of the study design and workflow. CASP7 = caspase-7, eQTL = expression quantitative trait locus, GEO = Gene Expression Omnibus, GWAS = genome-wide association study, mQTL = methylation quantitative trait locus, pQTL = protein quantitative trait locus, SMR = summary-based Mendelian randomization.

Figure 1 demonstrates the overall design of this study. QTL data, GWAS, and GEO datasets were publicly available (Table S1, Supplemental Digital Content 1). All included studies received approval from their respective institutional review boards.

### 2.2. Data sources

#### 2.2.1. Outcome data sources

The GWAS data for age-related cataract and other cataract subtypes were obtained from *the OpenGWAS project database, the UK Biobank database, and the GWAS Catalog database.* These datasets are publicly accessible, and all participants included were of European ancestry. The sample sizes included: 49,250 cataract cases and 3,58,496 controls; 14,025 cataract cases and 1,33,954 controls; 7038 cataract cases and 4,87,560 controls; 18,988 cataract cases and 4,01,543 controls.

For this study, *the OpenGWAS database* served as the discovery stage, while *the OpenGWAS database*, *the UK Biobank database,* and *the GWAS Catalog database* were used as the replication stages. Different subtypes of cataracts were analyzed for validation.

#### 2.2.2. QTL data sources

Three primary QTL –datasets – mQTL, eQTL, and pQTL – were utilized in this study. mQTL data were derived from McRae et al, who analyzed SNP-CpG associations in blood samples from 1980 individuals of European ancestry.^[19]^ eQTL data were obtained from the eQTLGen Consortium, which included large-scale blood expression data from 31,684 individuals.^[20]^ pQTL data were obtained from Ferkingstad et al, who analyzed circulating proteins in 35,559 Icelandic individuals.^[21]^ These blood-derived QTL resources were selected because they are large, publicly available, well-powered, and ancestry-compatible with the cataract GWAS datasets. This discovery-stage use of blood-derived QTL resources is consistent with published multi-omics SMR studies that used large blood eQTL or mQTL resources for genetic prioritization and then incorporated tissue-relevant datasets for biological interpretation.^[14-17]^ We acknowledge that cataract is a lens-specific disease and that blood-based QTLs may not fully capture lens epithelial cell regulation; therefore, these QTL analyses should be interpreted as genetic prioritization rather than direct evidence of lens-specific regulation.

Additionally, tissue-specific eQTL data from the Genotype-Tissue Expression (GTEx) project were used as supportive context for tissue-specific expression patterns of target genes.^[22]^ Because publicly available lens-specific QTL resources are limited, whole-blood QTL data were used as the primary QTL source, and this tissue-context limitation was considered in the Discussion. Recent multi-context MR work further emphasizes that tissue context should be considered when interpreting molecular QTL-based prioritization.^[23]^

### 2.3. Mendelian randomization (MR) methods

Mendelian randomization (MR) uses single nucleotide polymorphisms (SNPs) as instrumental variables to evaluate genetically predicted associations between molecular exposures and disease outcomes. In this study, summary-data-based Mendelian randomization (SMR) was used to integrate mQTL, eQTL, and pQTL summary statistics with cataract GWAS data and to identify molecular traits associated with cataract risk. The SMR and HEIDI analyses were performed using SMR software version 1.3.1.

For the initial SMR screening, associations with *P*_SMR < .05 were considered candidate signals. To distinguish associations consistent with a shared causal variant from those potentially driven by linkage disequilibrium, the HEIDI test was applied. Signals with *P*_HEIDI ≤ .01 were considered to show evidence of heterogeneity consistent with linkage and were excluded from further interpretation, whereas signals with *P*_HEIDI > .01 were retained. To account for multiple testing, *P* values from the SMR analyses were further adjusted using the Benjamini–Hochberg false discovery rate (FDR) method.^[24]^ Candidate signals were then prioritized based on consistency across omics layers, FDR-adjusted significance, HEIDI filtering, and colocalization evidence where available. For colocalization analysis, signals with posterior probability for a shared causal variant (PPH4) > 0.70 were considered to provide supportive evidence of colocalization.

### 2.4. Colocalization analysis

Colocalization analysis was conducted to assess whether the identified mQTL, eQTL, or pQTL signals shared regional genetic evidence with cataract GWAS signals.^[25]^ Five mutually exclusive hypotheses were examined in the colocalization analysis: The variant is unrelated to any trait (H0). The variant is associated only with trait 1 (H1). The variant is associated only with trait 2 (H2). The causal variants for the 2 traits are different (H3). The causal variants for the 2 traits are the same (H4). The colocalization region windows were set as follows: ±1000 kb for pQTL-GWAS, ±1000 kb for eQTL-GWAS, and ±500 kb for mQTL-GWAS. A posterior probability (PPH4) >0.70 was considered strong evidence for colocalization.

### 2.5. Differential gene expression analysis using GEO

Gene expression data related to cataracts were obtained from the GEO database (http://www.ncbi.nlm.nih.gov/geo). We selected datasets derived from cataract-related rat lens tissue or lens epithelial cells because they provided a disease-relevant transcriptomic context for evaluating SMR-prioritized genes. The selected datasets included hereditary/spontaneous cataract and cataract-progression rat lens models, which are conceptually relevant to genetic-regulation-based prioritization but are not direct human age-related cataract samples. These datasets were used as orthogonal transcriptomic support rather than direct validation of human age-related cataract causality. Three datasets were selected: GSE230320, GSE230322, and GSE152616. The selected datasets used the GPL17117 platform and had compatible expression matrices and sample annotations. After merging the compatible expression matrices, batch effects among GEO datasets were corrected using the removeBatchEffect function from the limma R package.^[26]^ After batch correction, 18 samples were included, comprising 6 control samples and 12 cataract-model samples. Differential gene expression analysis was conducted on the batch-corrected matrix using the limma R package, with the following thresholds: |log_2_FC| ≥ 0.8 and adjusted *P* value < .05.

### 2.6. GO and KEGG enrichment analysis of shared DEGs

Shared DEGs were subjected to Gene Ontology (GO) functional enrichment analysis and Kyoto Encyclopedia of Genes and Genomes (KEGG) pathway analysis using the clusterProfiler R package. GO functional annotations included 3 categories: biological process (BP), cellular component (CC), and molecular function (MF). Statistically significant pathways or functional annotations were identified using a multiple-correction threshold of *P* < .05.

## 3. Results

### 3.1. Gene methylation and cataracts

**Figure 2.**
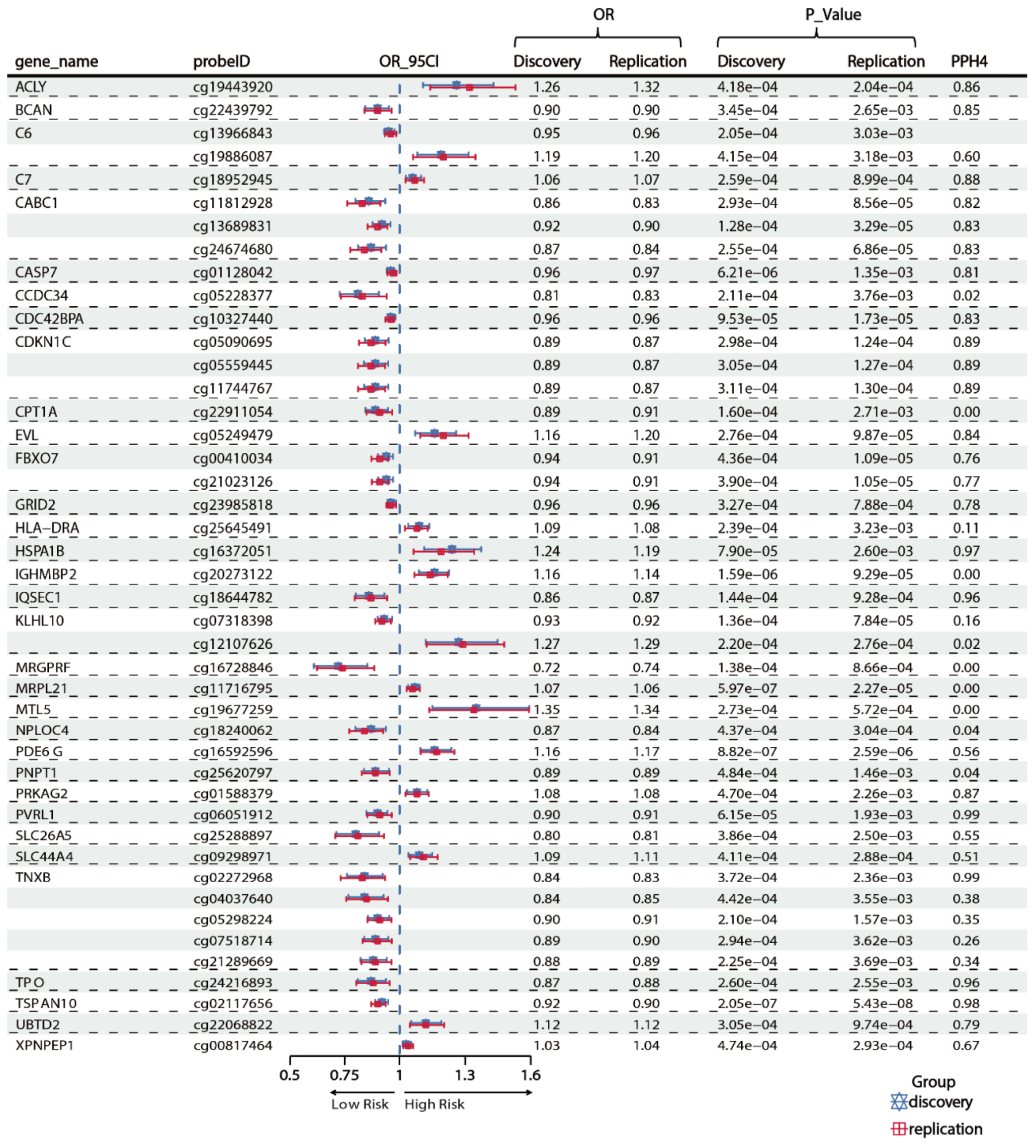
Associations of gene methylation with cataract in discovery and replication stages. CI = confidence interval, OR = odds ratio, PPH4 = posterior probability of H4.

Figure 2 demonstrates the association between DNA methylation and cataract, as well as validation results in independent stages. After initial SMR screening and HEIDI filtering, 671 methylation sites in the discovery stage and 622 methylation sites in the replication stage were identified (Table S2, Supplemental Digital Content 2 and Table S3, Supplemental Digital Content 3). The integration of discovery and replication stages further revealed 31 methylation sites located within 25 genes, and the complete list is provided in the supplementary tables to improve the readability of the main text. CASP7 cg01128042 showed a protective-direction association in the discovery analysis (OR = 0.958, 95% CI = 0.941–0.976, *P*_SMR = 6.21 × 10^−6^, FDR-adjusted *P* = .039, *P*_HEIDI = .440) and a consistent direction in the replication analysis (OR = 0.966, 95% CI = 0.946–0.987, *P*_SMR = .00135, *P*_HEIDI = .139). Methylated genes including CASP7, FBXO7, MRGPR, NPLOC4, PDE6, and TPCN were further evaluated in age-related cataract and subtype datasets (Tables S4 and S5, Supplemental Digital Content 4).

### 3.2. Gene expression and cataracts

**Figure 3.**
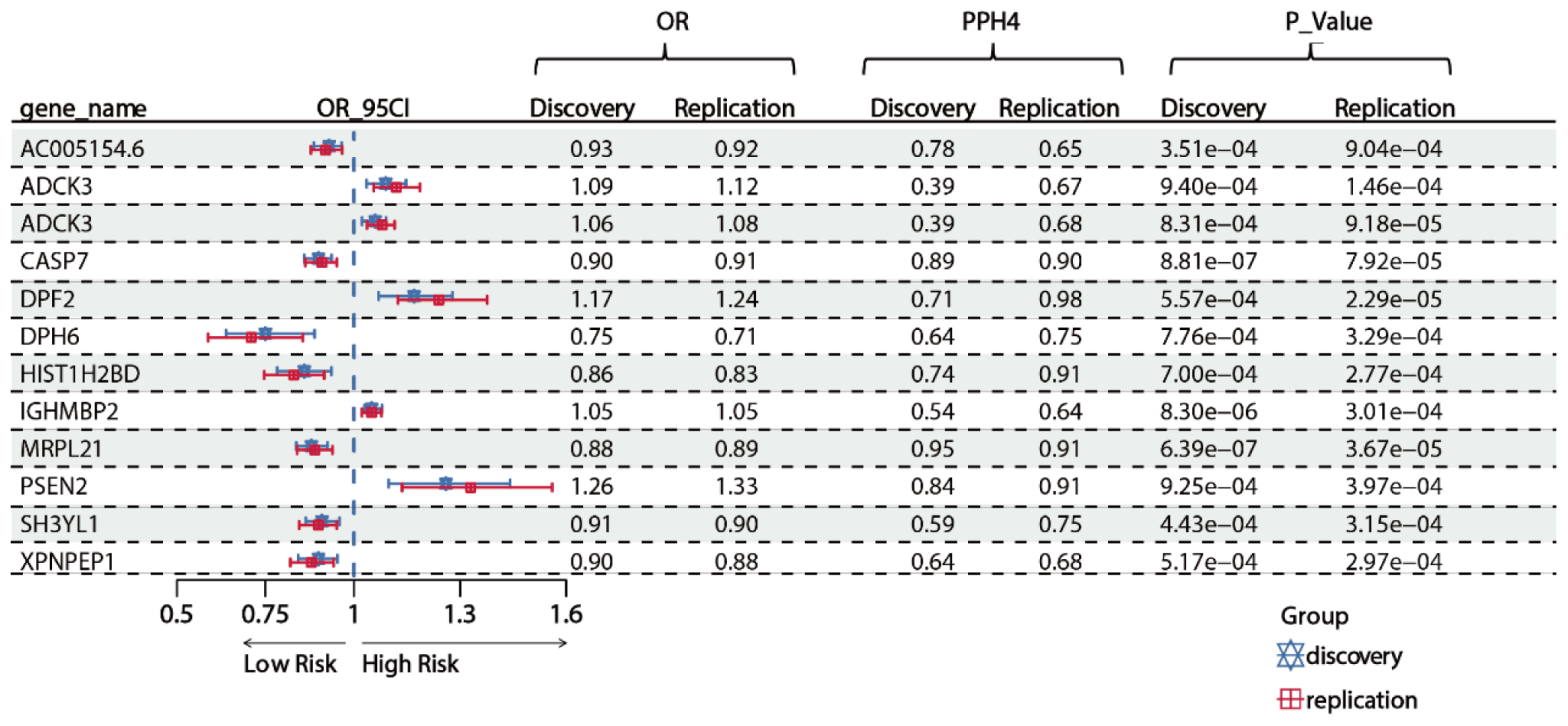
Associations of gene expression with cataract in discovery and replication stages. CI = confidence interval, OR = odds ratio, PPH4 = posterior probability of H4.

The gene-expression SMR results for cataract in the discovery and replication stages are shown in Figure 3. After initial screening using *P*_SMR < .05 and *P*_HEIDI > .01, 214 and 160 genes were identified as candidate signals in the discovery and replication stages, respectively (Table S6, Supplemental Digital Content 5 and Table S7, Supplemental Digital Content 6). Following FDR correction and colocalization analysis, CASP7, MRPL21, DPF2, HIST1H2BD, PSEN2, AC005154.6, and XPNPEP1 were prioritized as candidate genes associated with cataract. CASP7 showed a protective-direction estimate in the discovery eQTL analysis (OR = 0.897, 95% CI = 0.860–0.937, *P*_SMR = 8.81 × 10^−7^, FDR-adjusted *P* = .0046, *P*_HEIDI = .344) and a consistent direction in replication (OR = 0.906, 95% CI = 0.863–0.952, *P*_SMR = 7.92 × 10^−5^, *P*_HEIDI = 0.296). Colocalization supported a shared regional signal for CASP7 in this layer (PPH4 approximately 0.89 in discovery and 0.90 in replication).

Additionally, CASP7 and XPNPEP1 were validated in both age-related cataract and other cataract subtypes (Tables S8 and S9, Supplemental Digital Content 7). The data on different types of cataracts were obtained from *The UK Biobank* database and *The GWAS Catalog database* (Table S1, Supplemental Digital Content 1).

### 3.3. Protein expression and cataracts

**Figure 4.**
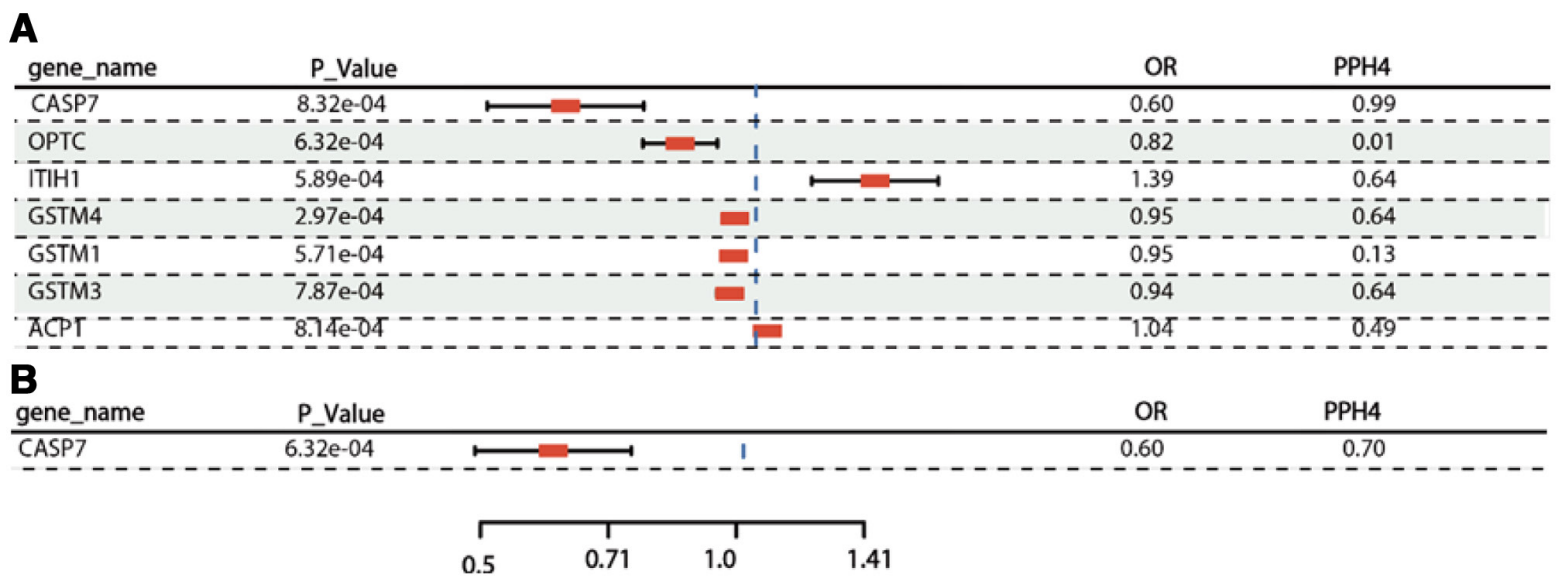
Associations of protein expression with cataract in discovery and replication stage. (A) Associations of protein expression with cataract in discovery stage. (B) Associations of protein expression with cataract in replication stage. CI = confidence interval, OR = odds ratio, PPH4 = posterior probability of H4.

The protein-level SMR results for cataract in the discovery and replication stages are presented in Figure 4. After initial screening using *P*_SMR < .05 and *P*_HEIDI > .01, 193 and 178 proteins were identified as candidate signals in the discovery and replication stages, respectively (Table S10, Supplemental Digital Content 8 and Table S11, Supplemental Digital Content 9). CASP7 showed the strongest protective-direction protein signal in the discovery analysis (OR = 0.597, 95% CI = 0.483–0.738, *P*_SMR = 1.85 × 10^−6^, FDR-adjusted *P* = .00083, *P*_HEIDI = .481), with directionally consistent evidence in the replication analysis (OR = 0.617, 95% CI = 0.487–0.782, *P*_SMR = 6.54 × 10^−5^, FDR-adjusted *P* = .029, *P*_HEIDI = .554). Other proteins with supportive signals included OPTC, ITIH1, GSTM4, GSTM1, GSTM3, ACP1, and LAYN. CASP7 and XPNPEP1 were further evaluated in age-related cataract and other subtype datasets (Tables S12 and S13, Supplemental Digital Content 10).

### 3.4. Integration SMR analysis results

**Figure 5.**
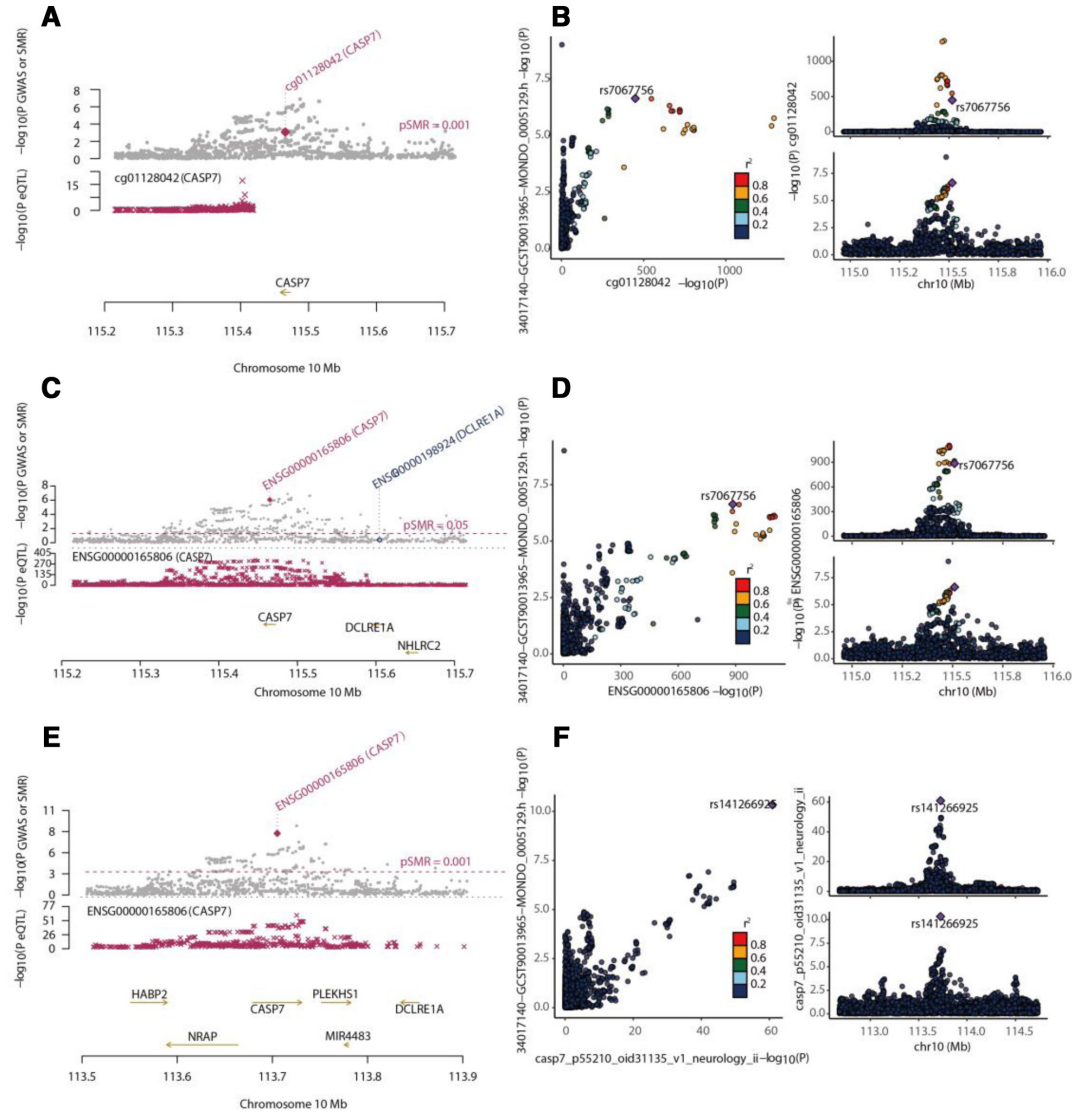
Visualization of SMR and colocalization analyses for related genes combined with SMR methylation, gene expression, and protein data. (A) Chromosomal Manhattan plot and colocalization analysis for the association between CASP7 methylation and cataract GWAS identified through SMR analysis. (B) Colocalization analysis for the association between CASP7 methylation and cataract GWAS identified through SMR analysis. (C) Chromosomal Manhattan plot for the association between CASP7 gene expression and cataract GWAS identified through SMR analysis. (D) Colocalization analysis for the association between CASP7 gene expression and cataract GWAS identified through SMR analysis. (E) Chromosomal Manhattan plot for the association between CASP7 protein levels and cataract GWAS identified through SMR analysis. (F) Colocalization analysis for the association between CASP7 protein levels and cataract GWAS identified through SMR analysis. CASP7 = caspase-7, eQTL = expression quantitative trait locus, GWAS = genome-wide association study, SMR = summary-based Mendelian randomization.

Following integration of mQTL, eQTL, and pQTL SMR results, CASP10, XPNPEP1, AGER, and CASP7 showed evidence across the 3 omics layers before final filtering. After multiple-testing correction and colocalization filtering (PPH4 > 0.70 where available), CASP7 remained the most consistent cross-omics candidate (Fig. 5). In the SMR framework, OR < 1 indicates that genetically predicted higher CASP7-related molecular exposure was associated with lower cataract odds. Because the exposure scales differ across methylation, expression, and protein QTL datasets, these ORs should be interpreted within each omics layer rather than directly compared as equivalent biological effect sizes. The integrated estimates were mQTL: OR = 0.959 (95% CI = 0.941–0.977, FDR-adjusted *P* = .039), eQTL: OR = 0.897 (95% CI = 0.860–0.937, FDR-adjusted *P* = .004), and pQTL: OR = 0.597 (95% CI = 0.483–0.738, FDR-adjusted *P* = .00083).

### 3.5. GEO analyses

**Figure 6.**
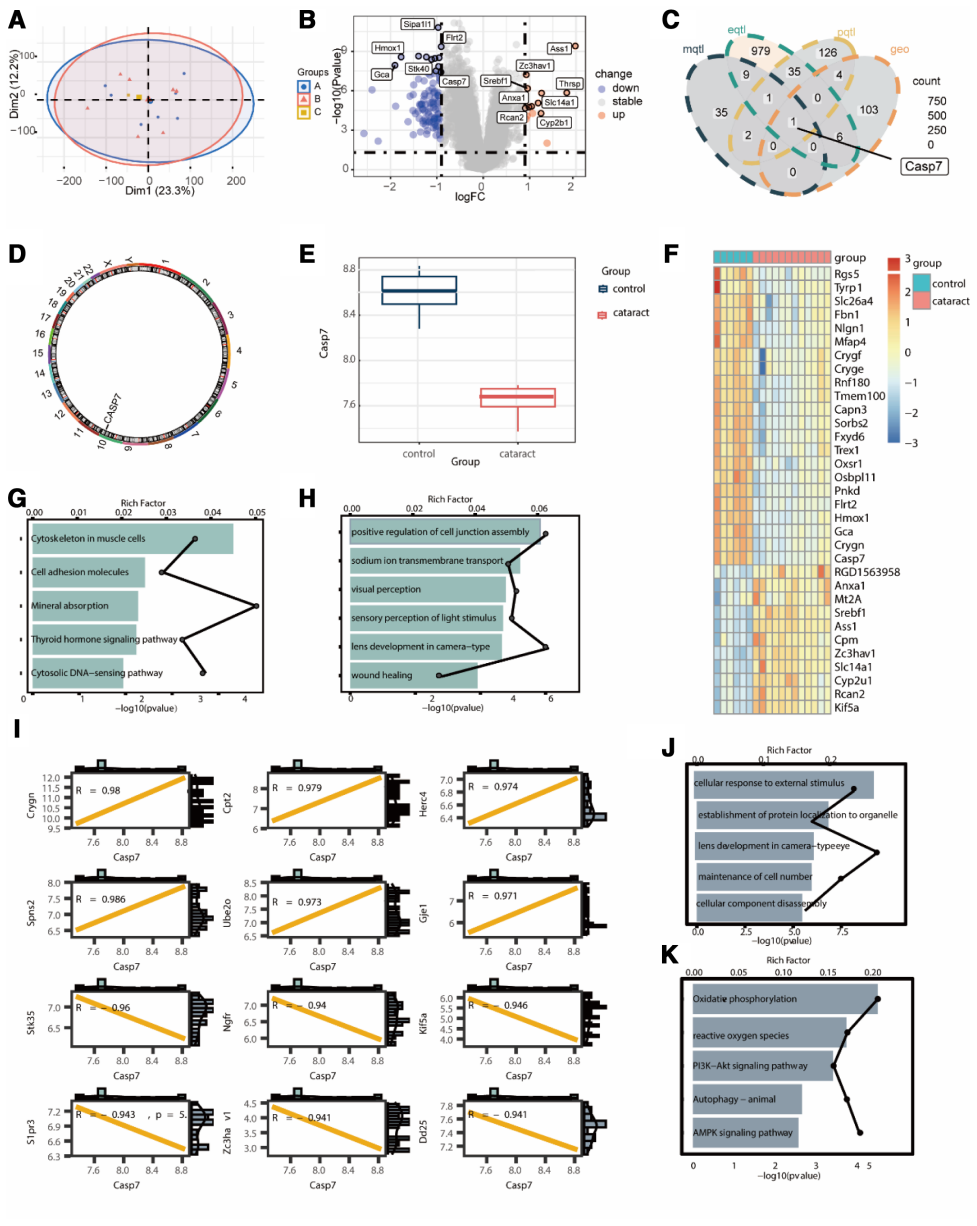
GEO analyses. (A) Batch effect correction of 3 datasets. (B) Volcano plot of differentially expressed genes. (C) Venn diagrams of the integration of the results of SMR and DEGs. (D) Circular map of CASP7 chromosome. (E) Box plot of differential expression of CASP7 between cataract and normal group. (F) Heatmap of differentially expressed genes between control and cataract groups. (G) KEGG enrichment analysis of differentially expressed genes. (H) GO enrichment analysis of differentially expressed genes. (I) Correlation analysis between CASP7 and other genes. (J) KEGG enrichment analysis of correlated genes. (K) GO enrichment analysis of correlated genes. CASP7 = caspase-7, DEG = database for differentially expressed gene, eQTL = expression quantitative trait locus, GEO = Gene Expression Omnibus, GO = Gene Ontology, KEGG = Kyoto Encyclopedia of Genes and Genomes pathway, mQTL = methylation quantitative trait locus, pQTL = protein quantitative trait locus, SMR = summary-based Mendelian randomization.

The datasets GSE230320, GSE230322, and GSE152616 were merged, resulting in 6 control samples and 12 cataract-model samples, including cataract-related lens models with cortical, nuclear, and posterior subcapsular opacity features.^[27,28]^ Batch effects were corrected using limma::removeBatchEffect, and the correction result is shown in Figure 6A. Differential gene expression analysis revealed 99 downregulated and 12 upregulated genes in cataract models compared with controls (Fig. 6B, F). Integrating SMR-prioritized candidates with GEO DEGs identified CASP7 as an overlapping gene (Fig. 6C), and CASP7 expression was lower in cataract-model samples than in controls (Fig. 6E). The chromosome circle diagram indicates that CASP7 is located on chromosome 10 (Fig. 6D). GO and KEGG enrichment analyses were conducted on the merged data. The cell adhesion molecules pathway was enriched in KEGG analysis, and GO analysis identified membrane repair-related pathways as significantly enriched, with CASP7 as one of the key genes in this process (Fig. 6G, H). Gene correlation analysis showed that Crygn exhibited the most positive correlation with CASP7, whereas Stk35 showed the most negative correlation (Fig. 6I). Genes correlated with CASP7 were enriched in cellular response to external stimulus and autophagy (Fig. 6J, K). These GEO results provide lens-related transcriptomic support for CASP7 but should not be interpreted as direct validation of human age-related cataract causality.

## 4. Discussion

In this study, we used DNA methylation, gene expression, and protein abundance QTL data as molecular exposure layers and cataract GWAS summary statistics as outcomes. By combining SMR, HEIDI filtering, and colocalization analysis, we prioritized CASP7 as a candidate protective gene associated with cataract risk. The convergence across methylation, expression, and protein layers strengthens genetic prioritization of CASP7. However, these analyses do not prove biological causation, and the GEO analysis should be considered transcriptomic support rather than functional validation.

CASP7 is traditionally considered an executioner caspase that partly overlaps with CASP3 in apoptosis.^[29]^ Recent studies have suggested additional roles for CASP7 in inflammatory cell-death regulation and membrane repair.^[30-32]^ For example, CASP7 can activate acid sphingomyelinase, which promotes ceramide production and facilitates repair of gasdermin and perforin pores.^[31,32]^ Pyroptosis and oxidative stress have been implicated in cataract-related lens epithelial cell injury.^[33-35]^ Therefore, the protective-direction CASP7 associations observed in this study are biologically plausible and are consistent with a hypothesis in which CASP7 may participate in stress–response or membrane-repair pathways in lens-related cells. Nevertheless, this mechanism remains inferential and requires direct experimental validation.

Several limitations should be considered. First, the study relied on publicly available summary-level and transcriptomic datasets, and no new functional experiments were performed. Second, the mQTL, eQTL, and pQTL resources were derived mainly from blood or circulating proteins. Although these resources provide statistical power and broad molecular coverage, they may not reflect lens epithelial cell-specific regulation. Third, the GEO datasets were derived from rat hereditary/spontaneous cataract or cataract-progression lens models, which differ from human age-related cataract in species, developmental context, and disease mechanism. Fourth, the GWAS and QTL datasets were largely of European ancestry. This reduces ancestry mismatch within the analysis but limits generalizability to other populations. Future studies should validate CASP7 in human lens-relevant cells, lens tissue, multi-ancestry datasets, and experimental cataract models.

## 5. Conclusions

By integrating multi-omics QTL resources with cataract GWAS data, this study prioritized CASP7 as a genetically supported candidate protective gene associated with cataract risk. The findings suggest that CASP7 may be involved in cataract-relevant stress-response and membrane-repair pathways, but they do not establish CASP7 as a confirmed causal gene or therapeutic target. Functional validation in human lens-relevant systems is needed to clarify its biological role in cataract development.

## Acknowledgments

We want to acknowledge the participants and investigators of the IEU Open GWAS, GWAS Catalog, FinnGen study and GEO databases.

## Author contributions

**Conceptualization:** Zhengxi Yuan, Xiangyu Ding, Jingqing Mu, Xia Hua.

**Funding acquisition:** Xia Hua, Jingqing Mu.

**Investigation:** Zhengxi Yuan, Jingqing Mu, Xia Hua.

**Methodology:** Zhengxi Yuan, Xiaolong Fang.

**Project administration:** Jingqing Mu.

**Resources:** Xia Hua.

**Software:** Zhengxi Yuan, Xiaolong Fang.

**Supervision:** Xiangyu Ding.

**Validation:** Zhengxi Yuan, Jingqing Mu, Xia Hua.

**Writing – original draft:** Zhengxi Yuan.

**Writing – review & editing:** Jingqing Mu, Xia Hua.


























